# Supplementary figures and images for: Plasma-based lipidomics reveals potential diagnostic biomarkers for esophageal squamous cell carcinoma: a retrospective study
Source: PeerJ. 2024 Apr 29;12:e17272. doi: 10.7717/peerj.17272 (PMC11064858; doi:10.7717/peerj.17272)

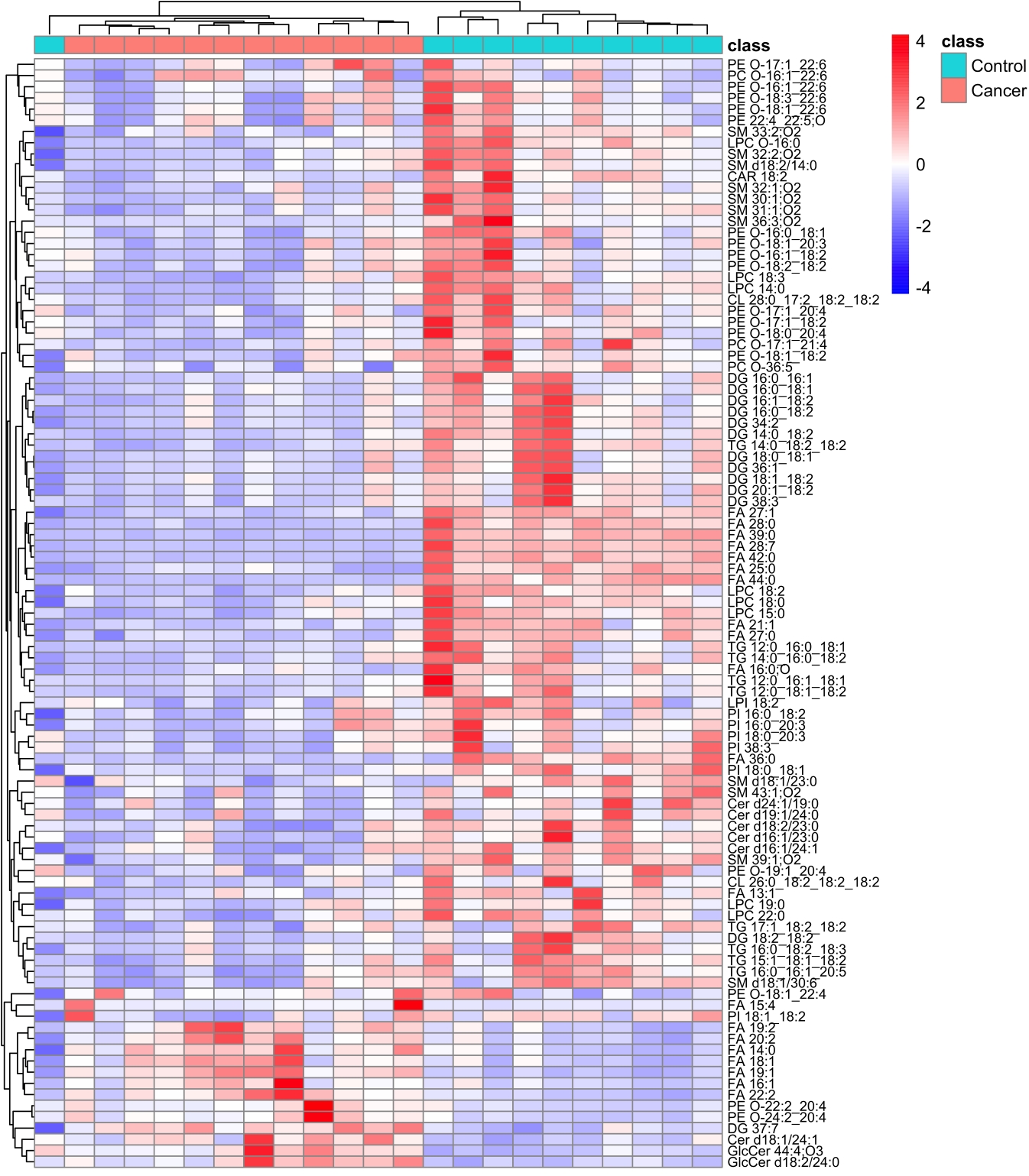

Supplement: Figure S1 [file peerj-12-17272-s001.jpg]

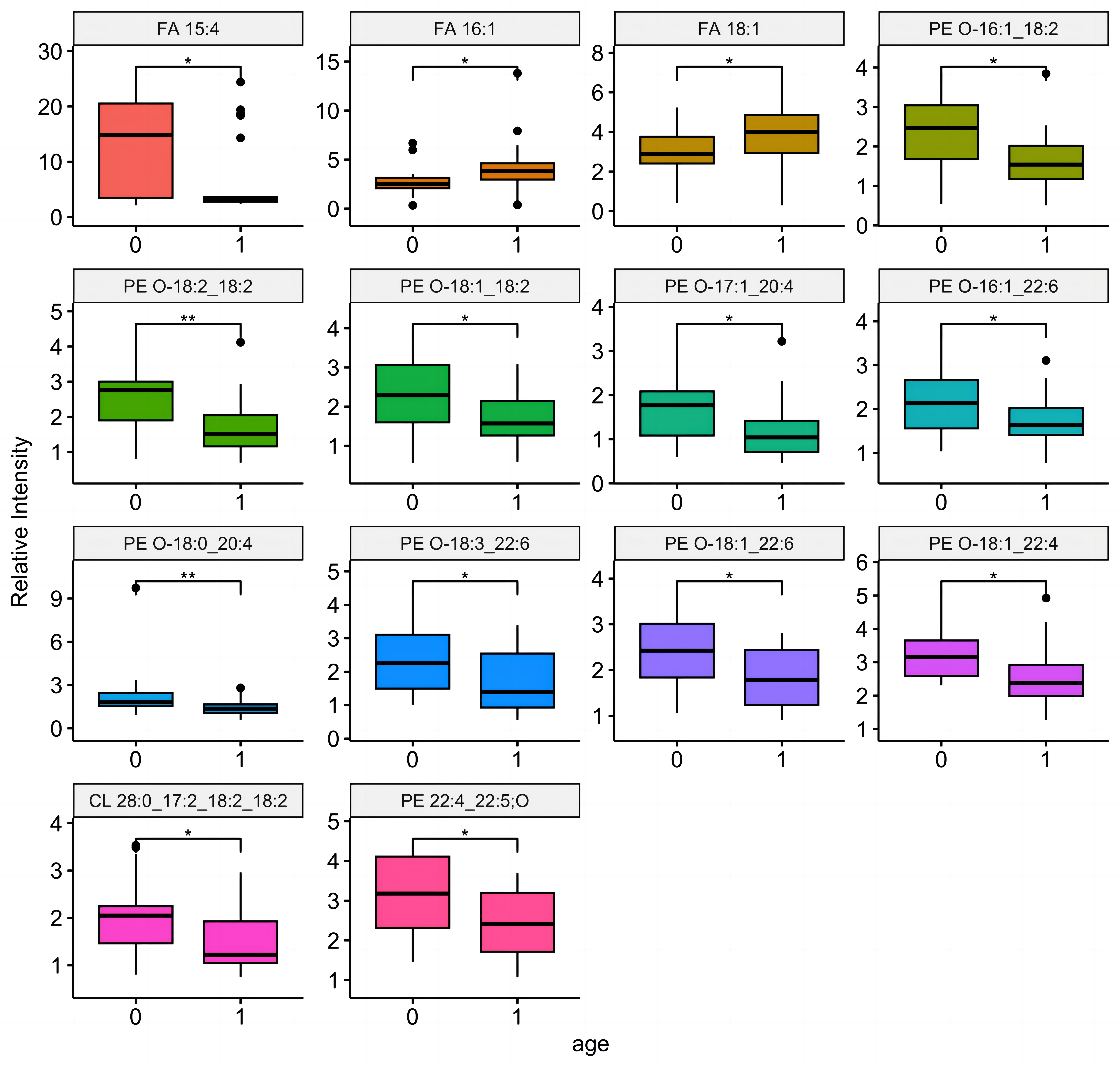

Supplement: Figure S2 — 0: the age of ESCC patients <60 years, 1: the age of ESCC patients ≥60 years. Wilcoxon test was performed to identify differential lipids associated with the factor of age in ESCC patients. Lipids were selected based on a significance threshold of P-value < 0.05. [file peerj-12-17272-s002.jpg]

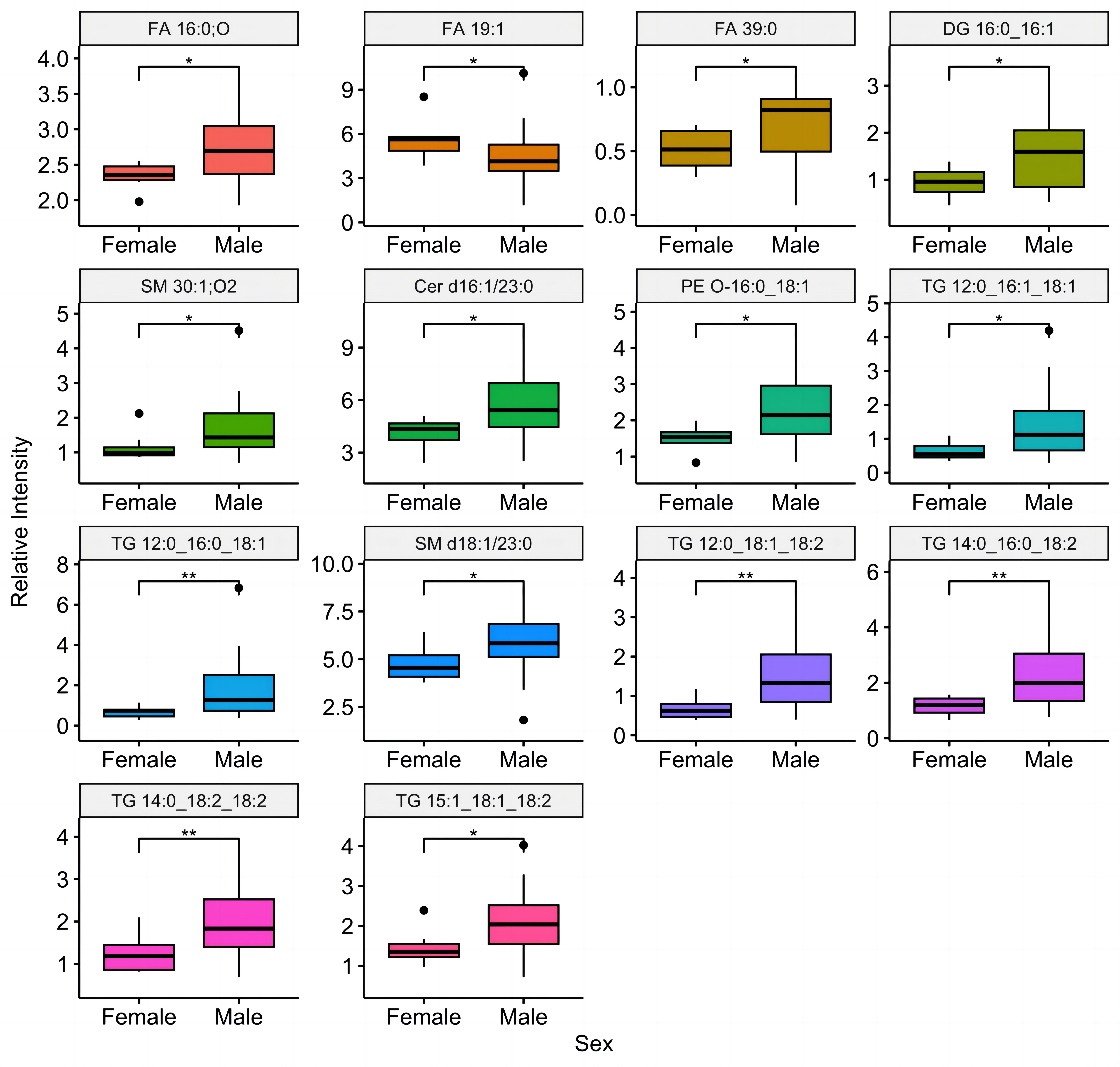

Supplement: Figure S3 — Wilcoxon test was performed to identify differential lipids associated with the factor of sex in ESCC patients. Lipids were selected based on a significance threshold of P-value < 0.05. [file peerj-12-17272-s003.jpg]

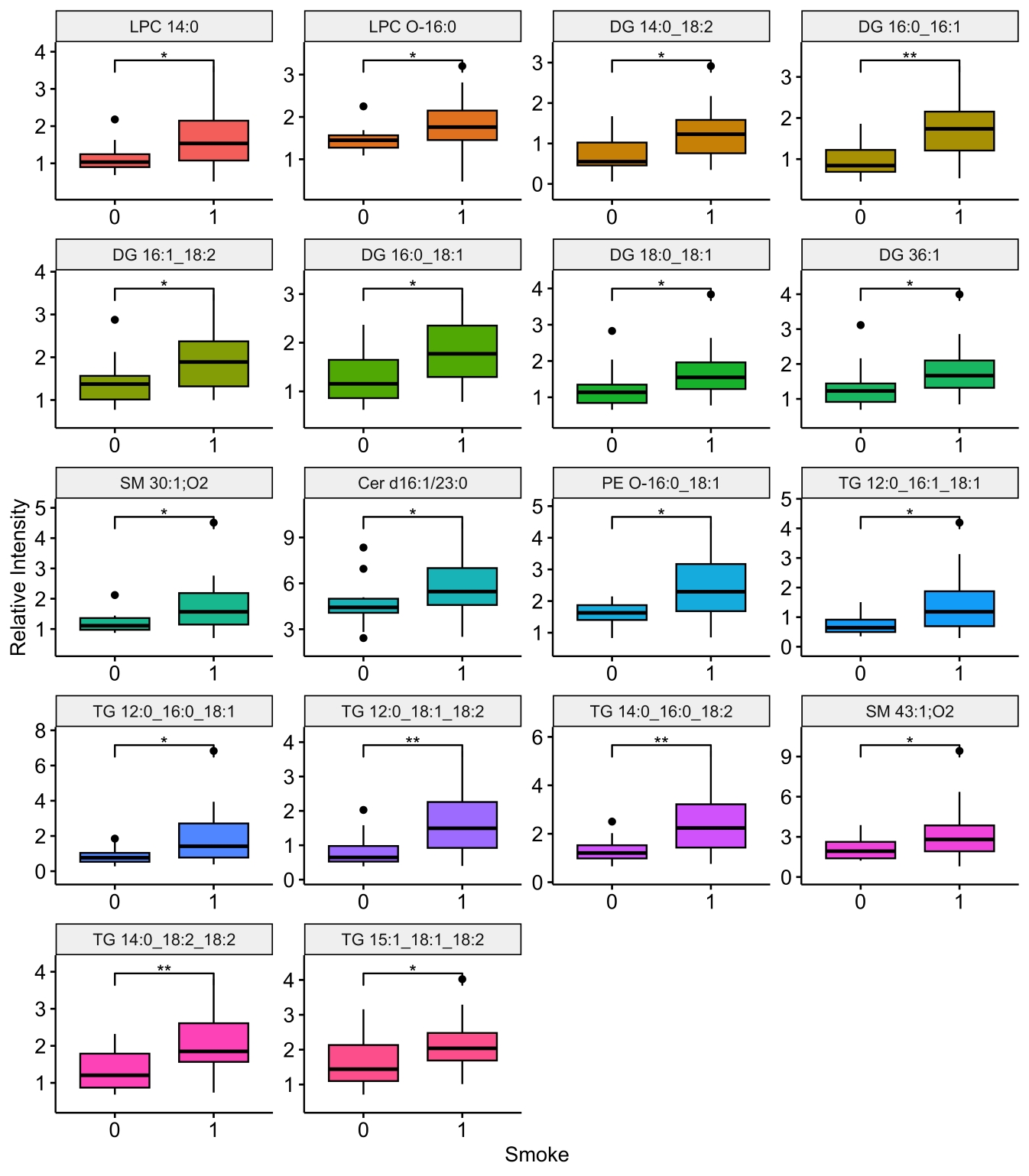

Supplement: Figure S4 — Wilcoxon test was performed to identify differential lipids associated with the factor of smoking in ESCC patients. 0: ESCC patients without smoking history, 1: ESCC patients with smoking history. Lipids were selected based on a significance threshold of P-value < 0.05. [file peerj-12-17272-s004.jpg]

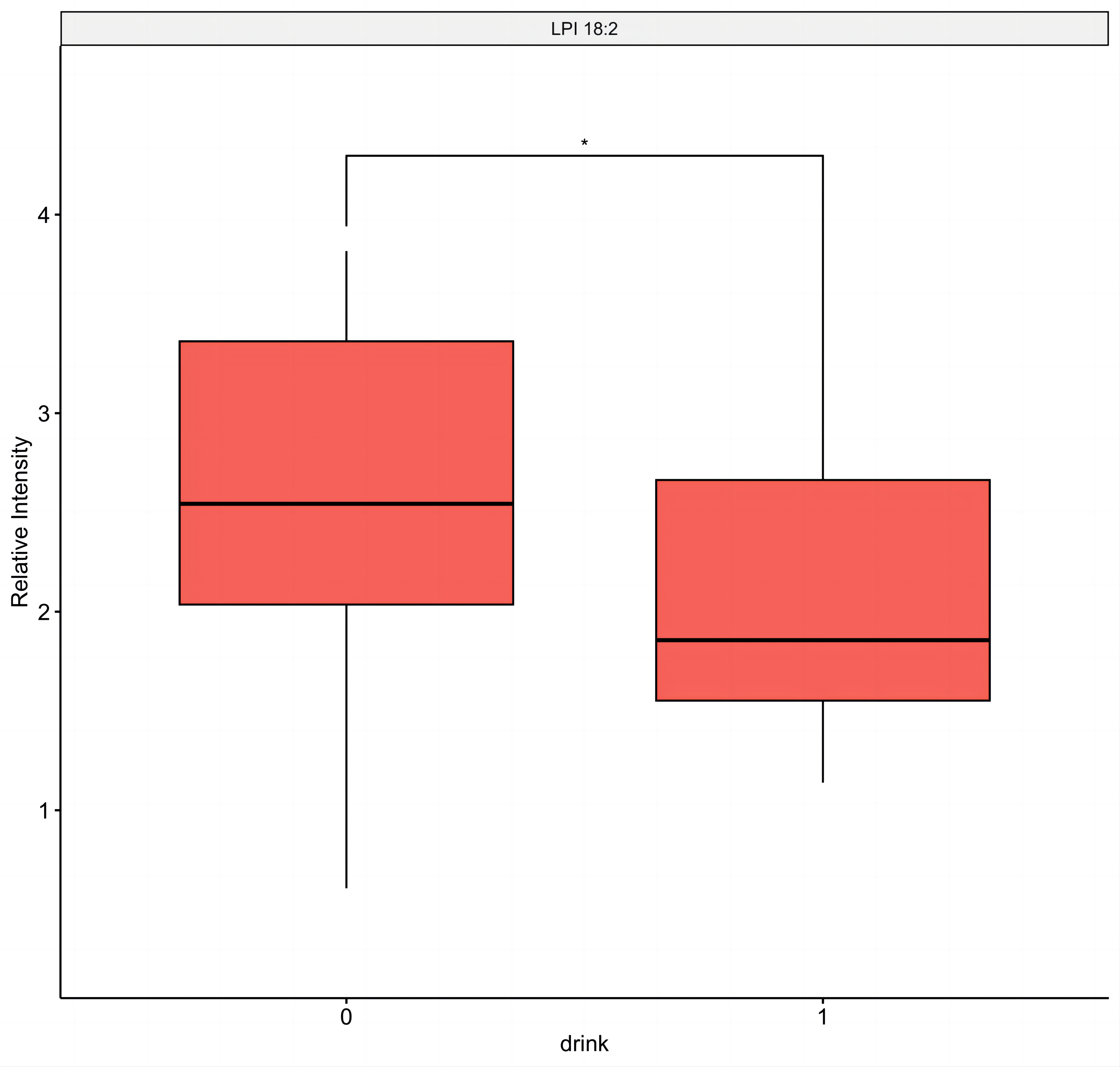

Supplement: Figure S5 — Wilcoxon test was performed to identify differential lipids associated with the factor of drinking in ESCC patients. 0: ESCC patients without drinking history, 1: ESCC patients with drinking history. Lipids were selected based on a significance threshold of P-value < 0.05. [file peerj-12-17272-s005.jpg]
