# Supplementary material for: Plasma-based lipidomics reveals potential diagnostic biomarkers for esophageal squamous cell carcinoma: a retrospective study
Source: PeerJ. 2024 Apr 29;12:e17272. doi: 10.7717/peerj.17272 (PMC11064858; doi:10.7717/peerj.17272)
Supplement: Supplemental Information 14 — Checklist of items that should be included in reports of case-control studies. [file peerj-12-17272-s014.docx]

STROBE Statement—checklist of items that should be included in reports of observational studies

|  | Item No. | Recommendation | Page  No. | Relevant text from manuscript |
| --- | --- | --- | --- | --- |
| **Title and abstract** | 1 | (*a*) Indicate the study’s design with a commonly used term in the title or the abstract | 2 | **Methods.** Plasma samples from a total of 40 ESCC patients and 31 healthy controls were used for lipidomics study. Untargeted lipidomics analysis was conducted through liquid chromatography-mass spectrometry (LC-MS) analysis. Differentially expressed lipid features were filtered based on multivariate and univariate analysis, and lipid annotation was performed using MS-DIAL software. |
|  |  | (*b*) Provide in the abstract an informative and balanced summary of what was done and what was found | 2 | **Results.** A total of 99 differential lipids were identified, with 15 up-regulated lipids and 84 down-regulated lipids, suggesting their potential as diagnostic targets for ESCC. In the single-lipid plasma-based diagnostic model, nine specific lipids (FA 15:4, FA 27:1, FA 28:7, FA 28:0, FA 36:0, FA 39:0, FA 42:0, FA 44:0, and DG 37:7) exhibited excellent diagnostic performance, with an area under the curve (AUC) exceeding 0.99. Furthermore, multiple lipid-based ML models also demonstrated comparable diagnostic ability for ESCC. These findings present plasma lipids as a promising diagnostic approach for ESCC. |
| Introduction | | | |  |
| Background/rationale | 2 | Explain the scientific background and rationale for the investigation being reported | 3 | Esophageal cancer (EC) is the eighth most prevalent malignancy in the world and the sixth leading cause of cancer-related death (Morgan et al. 2022). Histologically, EC can be classified into two distinct subtypes, esophageal squamous cell carcinoma (ESCC) and esophageal adenocarcinoma (EA). The former accounts for 90% of all cases and developing countries bear the burden of 80% of global cases (Liang et al. 2017). Ongoing research has identified alcohol abuse and smoking as the two most definitive risk factors for ESCC (Reichenbach et al. 2019), and other uncertain risk factors include radiation and pesticide exposure, sedentary lifestyle, and diet with low-fiber intake (Codipilly & Wang 2022). |
| Objectives | 3 | State specific objectives, including any prespecified hypotheses | 3 | Metabolic disorders, including carbohydrate metabolism, amino acid metabolism, nucleotide metabolism, and lipid metabolism, play crucial roles in tumorigenesis (Huang et al. 2020; Kaushik & DeBerardinis 2018; Schmidt et al. 2021). Currently, metabolomics has emerged as a powerful tool for identifying metabolic alterations in various disease (Li et al. 2021). Lipidomics, as a branch of metabolomics, has gained traction in cancer research due to the detection of dysregulated lipid metabolism in tumors, including ESCC (Liang et al. 2021; Yuan et al. 2021). |
| Methods | | | |  |
| Study design | 4 | Present key elements of study design early in the paper | 4-7 | Plasma samples from a total of 40 ESCC patients and 31 healthy controls were used for lipidomics study. Untargeted lipidomics analysis was conducted through liquid chromatography-mass spectrometry (LC-MS) analysis. Differentially expressed lipid features were filtered based on multivariate and univariate analysis, and lipid annotation was performed using MS-DIAL software. |
| Setting | 5 | Describe the setting, locations, and relevant dates, including periods of recruitment, exposure, follow-up, and data collection | 4 | This retrospective study analyzed plasma samples which were obtained from Zhejiang Cancer Hospital (Hangzhou, China) between December 2010 and December 2012. Plasma samples were collected from 40 pathologically diagnosed ESCC patients and 31 healthy controls (HCs). Table 1 presents the basic clinical information of the participants. Blood was collected from individuals who had fasted overnight and transferred into vials pre-treated with the anticoagulant reagent (ethylenediaminetetraacetic acid disodium potassium salt). Plasma was obtained by centrifuging the blood at 2400xg for 8 minutes. The samples were then stored at -80°C until analysis. All procedures involving human participants were conducted in accordance with the ethical standards set by the Ethics Committee of Zhejiang Cancer Hospital (IRB- 2019-66), following the principles of the 1964 Helsinki Declaration and its subsequent amendments or comparable ethical standards. Furthermore, since the samples used in our study came from the biobank, it was agreed that patient consent was not required, and relevant materials were provided as supplementary materials. |
| Participants | 6 | (*a*) *Cohort study*—Give the eligibility criteria, and the sources and methods of selection of participants. Describe methods of follow-up  *Case-control study*—Give the eligibility criteria, and the sources and methods of case ascertainment and control selection. Give the rationale for the choice of cases and controls  *Cross-sectional study*—Give the eligibility criteria, and the sources and methods of selection of participants | 4 | This retrospective study analyzed plasma samples which were obtained from Zhejiang Cancer Hospital (Hangzhou, China) between December 2010 and December 2012. Plasma samples were collected from 40 pathologically diagnosed ESCC patients and 31 healthy controls (HCs). Table 1 presents the basic clinical information of the participants. Blood was collected from individuals who had fasted overnight and transferred into vials pre-treated with the anticoagulant reagent (ethylenediaminetetraacetic acid disodium potassium salt). Plasma was obtained by centrifuging the blood at 2400xg for 8 minutes. The samples were then stored at -80°C until analysis. All procedures involving human participants were conducted in accordance with the ethical standards set by the Ethics Committee of Zhejiang Cancer Hospital (IRB- 2019-66), following the principles of the 1964 Helsinki Declaration and its subsequent amendments or comparable ethical standards. Furthermore, since the samples used in our study came from the biobank, it was agreed that patient consent was not required, and relevant materials were provided as supplementary materials. |
|  |  | (*b*) *Cohort study*—For matched studies, give matching criteria and number of exposed and unexposed  *Case-control study*—For matched studies, give matching criteria and the number of controls per case | 4 | This retrospective study analyzed plasma samples which were obtained from Zhejiang Cancer Hospital (Hangzhou, China) between December 2010 and December 2012. Plasma samples were collected from 40 pathologically diagnosed ESCC patients and 31 healthy controls (HCs). Table 1 presents the basic clinical information of the participants. Blood was collected from individuals who had fasted overnight and transferred into vials pre-treated with the anticoagulant reagent (ethylenediaminetetraacetic acid disodium potassium salt). |
| Variables | 7 | Clearly define all outcomes, exposures, predictors, potential confounders, and effect modifiers. Give diagnostic criteria, if applicable | 4 | This retrospective study analyzed plasma samples which were obtained from Zhejiang Cancer Hospital (Hangzhou, China) between December 2010 and December 2012. Plasma samples were collected from 40 pathologically diagnosed ESCC patients and 31 healthy controls (HCs). Table 1 presents the basic clinical information of the participants. Blood was collected from individuals who had fasted overnight and transferred into vials pre-treated with the anticoagulant reagent (ethylenediaminetetraacetic acid disodium potassium salt). |
| Data sources/ measurement | 8* | For each variable of interest, give sources of data and details of methods of assessment (measurement). Describe comparability of assessment methods if there is more than one group | 5 | LC-MS analysis was conducted according to previous research (Yang et al. 2022). In brief, ultimate 3000 UHPLC system coupled with Q Exactive orbitrap mass Spectrometer (both form Thermo Fisher Scientific, USA) was used for lipidomics analysis. Chromatographic separation was carried out on an ACQUITY UPLC BEH C18 column (2.1mm × 100mm, 1.8μm, Waters, USA). Solvent A consisted of a mixture of ACN/water (3:2, v/v) containing 0.1% (v/v) formic acid and 10 mM ammonium acetate, while solvent B was composed of IPA/ACN (9:1, v/v) with the same additives. The flow rate was 0.3 mL/min, and column temperature was set at 50℃. The elution condition was set at 0.0-1.5min, 32% B; 1.5min-15.5min, 32%-85% B; 15.5-15.6min, 85%-97% B; 15.6-18.0min, 97% B; 18.0-18.1min, 97%-32%B; 18.1-20.0min, 32%B. The settings for the mass spectrometer included a capillary voltage of 3.0 kV and a capillary temperature of 300°C. The sheath gas flow rate was set to 50 Arb. The auxiliary gas had a flow rate and temperature of 15 Arb and 320°C, respectively. The scan range was set at m/z 100−1200. The full scan MS had a resolution of 70,000 and an AGC target of 3×10^6^. The data-dependent MS/MS had a resolution of 17,500 and an AGC target of 1×10^5^. The normalized collision energy was set to 30, 40, and 50 eV, respectively.  The analytical procedure employed a full scan mode to collect data from all samples in the batch. For qualitative quality control (QC) samples, data-dependent acquisition (DDA) mode was utilized. To ensure consistent performance and accuracy during the analysis, QC samples were interspersed within the sample injection sequence. The sequence commenced with three consecutive QC samples, followed by the inclusion of one QC sample every ten samples. The sequence concluded with another three consecutive QC samples. This approach helped to correct for mass spectrometry signal fluctuations and maintain reliable data quality throughout the analysis. |
| Bias | 9 | Describe any efforts to address potential sources of bias | 4 | The pooled quality control (QC) plasma samples were generated by combining equal aliquots of plasma from each individual sample, which were then dispensed into 40μL volumes. The extraction process employed for these pooled samples was identical to that used for the individual sample pretreatment |
| Study size | 10 | Explain how the study size was arrived at | 4 | Plasma samples were collected from 40 pathologically diagnosed ESCC patients and 31 healthy controls (HCs). |

Continued on next page

| Quantitative variables | 11 | Explain how quantitative variables were handled in the analyses. If applicable, describe which groupings were chosen and why | 5-6 | Data analysis was performed according to existing articles (Yang et al. 2022) with some modification. Briefly, the research utilized ProteoWizard's msconvert tool (available at <https://proteowizard.sourceforge.io/download.html>) to convert RAW format data into mzXML format data. The R package *xcms* was then employed for detecting and extracting ion features, which included tasks such as peak picking and retention time correction. To correct signal shifts, the R package *statTarget* utilized QC-based random forest signal correction (QC-RFSC). Subsequently, ion feature filtration was conducted. In this step, variables were retained if they had a non-zero value in at least 80% of samples within any single group. However, variables in QC samples with a relative standard deviation (RSD) greater than 30% were excluded. Imputation was performed using the K-nearest neighbors (KNN) algorithm. Prior to chemometrics analysis, the detected ions in each sample belonging to the same class were normalized by setting the sum of their peak areas to 100,000. This rigorous approach ensured that only the most consistent and reliable features were retained for further analysis, thereby significantly improving the overall robustness and reliability of the lipidomics data.  Train set samples were used to discover the differentially expressed plasma lipids between ESCC and HC groups. Firstly, unsupervised principal component analysis (PCA) was employed to visualize the overall separation trend of all samples based on the ion features. Subsequently, a supervised partial least squares discriminant analysis (PLS-DA) was utilized to assess the classification ability of these ion features, yielding a variable importance in projection (VIP) value for each ion feature. Furthermore, the statistical significance of the ion features between the ESCC and HC groups was evaluated using a two-tailed Student's t-test, and the Benjaminii-Hochberg false discovery rate (FDR) was also calculated. The criteria for defining differentially expressed ion features were as follows: VIP > 1.0, adjusted p-value (FDR) < 0.05, and fold change (FC) greater than 1.50 or less than 0.667.  The annotation of lipids was as previously described (Tsugawa et al. 2020). Briefly, the RAW format data was first converted to the abf format using Abf converter (<https://www.reifycs.com/abfconverter/>). Then, MS-DIAL software ver.4.9 (<http://prime.psc.riken.jp/compms/index.html>) was used to perform feature detection on all ions with the following parameters: the tolerance of MS1 and MS2 were set 0.01Da and 0.025Da; identification score cut off was set 80%; in positive mode, [M + H]^+^, [M + NH4]^+^, [M + Na]^+^ and [M + H-H2O]^+^ were selected as the adduct types; in negative mode, [M - H]^-^ was selected as the adduct type; the retention time was tolerance set to 0.05 min and MS1 tolerance was set to 0.015 Da in all ions feature alignment option.  The differential lipids between ESCC and HC groups in both train set and test set were illustrated using heatmap. |
| --- | --- | --- | --- | --- |
| Statistical methods | 12 | (*a*) Describe all statistical methods, including those used to control for confounding | 5-6 | Data analysis was performed according to existing articles (Yang et al. 2022) with some modification. Briefly, the research utilized ProteoWizard's msconvert tool (available at <https://proteowizard.sourceforge.io/download.html>) to convert RAW format data into mzXML format data. The R package *xcms* was then employed for detecting and extracting ion features, which included tasks such as peak picking and retention time correction. To correct signal shifts, the R package *statTarget* utilized QC-based random forest signal correction (QC-RFSC). Subsequently, ion feature filtration was conducted. In this step, variables were retained if they had a non-zero value in at least 80% of samples within any single group. However, variables in QC samples with a relative standard deviation (RSD) greater than 30% were excluded. Imputation was performed using the K-nearest neighbors (KNN) algorithm. Prior to chemometrics analysis, the detected ions in each sample belonging to the same class were normalized by setting the sum of their peak areas to 100,000. This rigorous approach ensured that only the most consistent and reliable features were retained for further analysis, thereby significantly improving the overall robustness and reliability of the lipidomics data.  Train set samples were used to discover the differentially expressed plasma lipids between ESCC and HC groups. Firstly, unsupervised principal component analysis (PCA) was employed to visualize the overall separation trend of all samples based on the ion features. Subsequently, a supervised partial least squares discriminant analysis (PLS-DA) was utilized to assess the classification ability of these ion features, yielding a variable importance in projection (VIP) value for each ion feature. Furthermore, the statistical significance of the ion features between the ESCC and HC groups was evaluated using a two-tailed Student's t-test, and the Benjaminii-Hochberg false discovery rate (FDR) was also calculated. The criteria for defining differentially expressed ion features were as follows: VIP > 1.0, adjusted p-value (FDR) < 0.05, and fold change (FC) greater than 1.50 or less than 0.667.  The annotation of lipids was as previously described (Tsugawa et al. 2020). Briefly, the RAW format data was first converted to the abf format using Abf converter (<https://www.reifycs.com/abfconverter/>). Then, MS-DIAL software ver.4.9 (<http://prime.psc.riken.jp/compms/index.html>) was used to perform feature detection on all ions with the following parameters: the tolerance of MS1 and MS2 were set 0.01Da and 0.025Da; identification score cut off was set 80%; in positive mode, [M + H]^+^, [M + NH4]^+^, [M + Na]^+^ and [M + H-H2O]^+^ were selected as the adduct types; in negative mode, [M - H]^-^ was selected as the adduct type; the retention time was tolerance set to 0.05 min and MS1 tolerance was set to 0.015 Da in all ions feature alignment option.  The differential lipids between ESCC and HC groups in both train set and test set were illustrated using heatmap. |
|  |  | (*b*) Describe any methods used to examine subgroups and interactions | 5-6 | Data analysis was performed according to existing articles (Yang et al. 2022) with some modification. Briefly, the research utilized ProteoWizard's msconvert tool (available at <https://proteowizard.sourceforge.io/download.html>) to convert RAW format data into mzXML format data. The R package *xcms* was then employed for detecting and extracting ion features, which included tasks such as peak picking and retention time correction. To correct signal shifts, the R package *statTarget* utilized QC-based random forest signal correction (QC-RFSC). Subsequently, ion feature filtration was conducted. In this step, variables were retained if they had a non-zero value in at least 80% of samples within any single group. However, variables in QC samples with a relative standard deviation (RSD) greater than 30% were excluded. Imputation was performed using the K-nearest neighbors (KNN) algorithm. Prior to chemometrics analysis, the detected ions in each sample belonging to the same class were normalized by setting the sum of their peak areas to 100,000. This rigorous approach ensured that only the most consistent and reliable features were retained for further analysis, thereby significantly improving the overall robustness and reliability of the lipidomics data.  Train set samples were used to discover the differentially expressed plasma lipids between ESCC and HC groups. Firstly, unsupervised principal component analysis (PCA) was employed to visualize the overall separation trend of all samples based on the ion features. Subsequently, a supervised partial least squares discriminant analysis (PLS-DA) was utilized to assess the classification ability of these ion features, yielding a variable importance in projection (VIP) value for each ion feature. Furthermore, the statistical significance of the ion features between the ESCC and HC groups was evaluated using a two-tailed Student's t-test, and the Benjaminii-Hochberg false discovery rate (FDR) was also calculated. The criteria for defining differentially expressed ion features were as follows: VIP > 1.0, adjusted p-value (FDR) < 0.05, and fold change (FC) greater than 1.50 or less than 0.667.  The annotation of lipids was as previously described (Tsugawa et al. 2020). Briefly, the RAW format data was first converted to the abf format using Abf converter (<https://www.reifycs.com/abfconverter/>). Then, MS-DIAL software ver.4.9 (<http://prime.psc.riken.jp/compms/index.html>) was used to perform feature detection on all ions with the following parameters: the tolerance of MS1 and MS2 were set 0.01Da and 0.025Da; identification score cut off was set 80%; in positive mode, [M + H]^+^, [M + NH4]^+^, [M + Na]^+^ and [M + H-H2O]^+^ were selected as the adduct types; in negative mode, [M - H]^-^ was selected as the adduct type; the retention time was tolerance set to 0.05 min and MS1 tolerance was set to 0.015 Da in all ions feature alignment option.  The differential lipids between ESCC and HC groups in both train set and test set were illustrated using heatmap. |
|  |  | (*c*) Explain how missing data were addressed |  | There is no missing data. |
|  |  | (*d*) *Cohort study*—If applicable, explain how loss to follow-up was addressed  *Case-control study*—If applicable, explain how matching of cases and controls was addressed  *Cross-sectional study*—If applicable, describe analytical methods taking account of sampling strategy | 5-6 | Data analysis was performed according to existing articles (Yang et al. 2022) with some modification. Briefly, the research utilized ProteoWizard's msconvert tool (available at <https://proteowizard.sourceforge.io/download.html>) to convert RAW format data into mzXML format data. The R package *xcms* was then employed for detecting and extracting ion features, which included tasks such as peak picking and retention time correction. To correct signal shifts, the R package *statTarget* utilized QC-based random forest signal correction (QC-RFSC). Subsequently, ion feature filtration was conducted. In this step, variables were retained if they had a non-zero value in at least 80% of samples within any single group. However, variables in QC samples with a relative standard deviation (RSD) greater than 30% were excluded. Imputation was performed using the K-nearest neighbors (KNN) algorithm. Prior to chemometrics analysis, the detected ions in each sample belonging to the same class were normalized by setting the sum of their peak areas to 100,000. This rigorous approach ensured that only the most consistent and reliable features were retained for further analysis, thereby significantly improving the overall robustness and reliability of the lipidomics data.  Train set samples were used to discover the differentially expressed plasma lipids between ESCC and HC groups. Firstly, unsupervised principal component analysis (PCA) was employed to visualize the overall separation trend of all samples based on the ion features. Subsequently, a supervised partial least squares discriminant analysis (PLS-DA) was utilized to assess the classification ability of these ion features, yielding a variable importance in projection (VIP) value for each ion feature. Furthermore, the statistical significance of the ion features between the ESCC and HC groups was evaluated using a two-tailed Student's t-test, and the Benjaminii-Hochberg false discovery rate (FDR) was also calculated. The criteria for defining differentially expressed ion features were as follows: VIP > 1.0, adjusted p-value (FDR) < 0.05, and fold change (FC) greater than 1.50 or less than 0.667.  The annotation of lipids was as previously described (Tsugawa et al. 2020). Briefly, the RAW format data was first converted to the abf format using Abf converter (<https://www.reifycs.com/abfconverter/>). Then, MS-DIAL software ver.4.9 (<http://prime.psc.riken.jp/compms/index.html>) was used to perform feature detection on all ions with the following parameters: the tolerance of MS1 and MS2 were set 0.01Da and 0.025Da; identification score cut off was set 80%; in positive mode, [M + H]^+^, [M + NH4]^+^, [M + Na]^+^ and [M + H-H2O]^+^ were selected as the adduct types; in negative mode, [M - H]^-^ was selected as the adduct type; the retention time was tolerance set to 0.05 min and MS1 tolerance was set to 0.015 Da in all ions feature alignment option.  The differential lipids between ESCC and HC groups in both train set and test set were illustrated using heatmap. |
|  |  | (*e*) Describe any sensitivity analyses | 6-7 | In order to assess the diagnostic value of plasma lipids, receiver operating characteristic (ROC) curve analysis was performed for each differentially expressed lipid in the training set using the R package *pROC*. This analysis allowed calculation of the area under the curve (AUC) values. The top nine lipids with the highest AUC values in the ROC curve were selected as the most diagnostic plasma lipids.  To determine the optimal cutoff value in the training set, the threshold was set at the point where the Youden Index (Sensitivity + Specificity - 1) was maximized. This approach aims to find the threshold that maximizes the difference between the true positive rate (sensitivity) and the false positive rate (1 - specificity), striking a balance between sensitivity and specificity.  To evaluate the diagnostic value of the plasma lipids, the cutoff value determined in the training set was used to predict the classification of samples in the test set. The performance of each lipid was then assessed using a confusion matrix, which allowed calculation of diagnostic metrics such as sensitivity, specificity, and accuracy. By following this procedure, the study aimed to identify the most informative plasma lipids for diagnostic purposes and evaluate their performance in classifying samples in both the training and test sets.  In addition to single lipid-based diagnostic models, multiple lipid-based ML models were investigated, including partial least squares (PLS) and random forest (RF) from the *caret* package, and support vector machine (SVM) from the *e1071* package. ROC curves were plotted for each model using the training set, and their prediction performance was evaluated in the test set using confusion matrix calculations. |
| Results | | | | |
| Participants | 13* | (a) Report numbers of individuals at each stage of study—eg numbers potentially eligible, examined for eligibility, confirmed eligible, included in the study, completing follow-up, and analysed | 7 | This study enrolled 40 patients pathologically diagnosed ESCC and 31 healthy controls (HCs). Age and gender were matched between the two groups without any statistically significant difference, as shown in Table 1. Among the ESCC patients, 9 were in stage I, 15 were in stage II, 16 were in stage III while no patients were in stage IV. The samples were randomly divided into a train set (ESCC = 28, HC = 20) and a test set (ESCC = 12, HC = 11). |
|  |  | (b) Give reasons for non-participation at each stage |  |  |
|  |  | (c) Consider use of a flow diagram |  |  |
| Descriptive data | 14* | (a) Give characteristics of study participants (eg demographic, clinical, social) and information on exposures and potential confounders | 7 | This study enrolled 40 patients pathologically diagnosed ESCC and 31 healthy controls (HCs). Age and gender were matched between the two groups without any statistically significant difference, as shown in Table 1. Among the ESCC patients, 9 were in stage I, 15 were in stage II, 16 were in stage III while no patients were in stage IV. The samples were randomly divided into a train set (ESCC = 28, HC = 20) and a test set (ESCC = 12, HC = 11). |
|  |  | (b) Indicate number of participants with missing data for each variable of interest | 7 | This study enrolled 40 patients pathologically diagnosed ESCC and 31 healthy controls (HCs). Age and gender were matched between the two groups without any statistically significant difference, as shown in Table 1. Among the ESCC patients, 9 were in stage I, 15 were in stage II, 16 were in stage III while no patients were in stage IV. The samples were randomly divided into a train set (ESCC = 28, HC = 20) and a test set (ESCC = 12, HC = 11). |
|  |  | (c) *Cohort study*—Summarise follow-up time (eg, average and total amount) |  |  |
| Outcome data | 15* | *Cohort study*—Report numbers of outcome events or summary measures over time |  |  |
|  |  | *Case-control study—*Report numbers in each exposure category, or summary measures of exposure | 7 | This study enrolled 40 patients pathologically diagnosed ESCC and 31 healthy controls (HCs). Age and gender were matched between the two groups without any statistically significant difference, as shown in Table 1. Among the ESCC patients, 9 were in stage I, 15 were in stage II, 16 were in stage III while no patients were in stage IV. The samples were randomly divided into a train set (ESCC = 28, HC = 20) and a test set (ESCC = 12, HC = 11). |
|  |  | *Cross-sectional study—*Report numbers of outcome events or summary measures |  |  |
| Main results | 16 | (*a*) Give unadjusted estimates and, if applicable, confounder-adjusted estimates and their precision (eg, 95% confidence interval). Make clear which confounders were adjusted for and why they were included | 8 | A total of 99 differentially expressed plasma lipids were identified and found to exhibit significant differences between individuals from ESCC and HC. Among these lipids, 15 were upregulated (FC > 1.5) and 84 were downregulated (FC < 0.667) in the plasma of ESCC patients compared to HC. The detailed information of these lipids is summarized in Table 2. Heatmap displaying the expression patterns of the differential lipids in the train set (Fig. 2A) revealed evident differences between ESCC and HC. Among the differential lipids, approximately three-fourths exhibited a downward trend in ESCC patients compared to HC. Similar expression patterns were observed in the test set (Supplemental Fig. S1). The proportion of lipid classifications was presented in the pie chart (Fig. 2B), including 21 fatty acids (FAs), 22 glycerolipids (GLs), 37 glycerophospholipids (GPs), 18 sphingolipids (SPs), and 1 sphingomyelin (SM). GPs represented the largest proportion of differential lipids at 37%, with 34 downregulated lipids and 3 upregulated lipids. Among the upregulated differential lipids (Fig. 2C), there were 8 FAs, 3 GPs and SPs, and 1 GL. Among the downregulated differential lipids (Fig. 2D), there were 13 FAs, 21 GLs, 34 GPs, 15 SPs, and 1 SM. |
|  |  | (*b*) Report category boundaries when continuous variables were categorized | 8 | ROC curve analysis revealed that plasma lipids show promise as diagnostic biomarkers for ESCC. The top nine lipids with the highest AUC values were FA 15:4, FA 27:1, FA 28:7, FA 28:0, FA 36:0, FA 39:0, FA 42:0, FA 44:0, and DG 37:7. Eight of these lipids achieved an AUC value of 1.00, indicating excellent diagnostic accuracy. FA 15:4 and DG 37:7 showed an up-regulated trend in ESCC samples, while the remaining lipids showed a down-regulated trend. (Fig. 3)  In testing step, the confusion matrix charts (Fig. 4) were constructed for a more intuitive representation of the diagnostic performance of the top nine differential lipids. All nine lipids depicted in the chart exhibited diagnostic performance exceeding 85%, with six exceeding 95%, and seven achieving 100% diagnostic efficiency in detecting tumors. The top 15 lipids, ranked by their prediction accuracy in the test set, were selected. Table 3 presents their AUC values in the train set, as well as prediction accuracy, sensitivity, specificity, precision, and recall values in the test set. Among these lipids, 13 belonged to FAs, and 11 lipids (FA 27:1, FA 28:7, FA 28:0, FA 39:0, FA 42:0, FA 44:0, FA 22:2, FA 36:0, FA 25:0, FA 19:1, FA 27:0) achieved prediction accuracy exceeding 0.90. The results for the remaining differential lipids can be found in the supplemental table S1.  Multiple lipid-based models were constructed using the training set data. The ROC curves in Figure 5A, B, C showed high AUC values of 0.990, 0.990, and 0.980, indicating excellent predictive performance. These results were consistent with the performance of individual differential lipids. The accuracy of the models was further validated in the test set, achieving accuracies of 95.7% (Fig. 5D, E, F). These findings demonstrate the effectiveness of the multiple lipid-based models as accurate diagnostic tools for ESCC. |
|  |  | (*c*) If relevant, consider translating estimates of relative risk into absolute risk for a meaningful time period | 9 | The results revealed that 14 differentially expressed plasma lipids were associated with age (Supplemental Fig. S2), 14 ones with sex (Supplemental Fig. S3), 18 ones with smoking history (Supplemental Fig. S4), and only one with drinking history (Supplemental Fig. S5).  Compared the ESCC patients with and without lymph node metastasis, there were 11 differential lipids showed significant difference (P-value < 0.05), including FA 14:0, FA 15:4, FA 27:1, FA 28:7, FA 27:0, FA 42:0, DG 37:7, PC O-16:1_22:6, PC O-17:1_21:4, SM d18:1/23:0, PI 18:0_20:3. Among them, 6 were up regulation and 5 were down regulation in ESCC patients with lymph node metastasis. Boxplots illustrating the expression levels of these 11 lipids were shown in Figure 6. Out of the 99 differential lipids, 10 lipids—FA 14:0, FA 15:4, FA 28:7, FA 27:0, FA 28:0, DG 37:7, PE O-17:1_22:6, PE O-18:3_22:6, PC O-16:1_22:6, and SM d18:1/23:0—exhibited significant differences among the three stage groups (P-value < 0.05). Boxplots depicting the expression levels of these 10 lipids are shown in Figure 7. Among them, FA 15:4 displayed the most prominent upregulated trend in stage III compared to stages I and II, with the P-value of 7.27e-07. Only one of these 10 lipids, PE O-18:3_22:6, exhibited statistically significant differences in pairwise comparisons across stages I, II, and III, with an increase in expression levels in stage II but declining in stage III patients. |

Continued on next page

| Other analyses | 17 | Report other analyses done—eg analyses of subgroups and interactions, and sensitivity analyses | 7 | Metabolic features were analyzed in train set and depicted in Figure 1. Following peak picking, retention time alignment, grouping, and signal shift correction, a total of 41028 ion features were obtained, including 22591 positive ions and 18437 negative ions. Based on these metabolic features, PCA and PLS-DA score plots were generated to investigate the differences between ESCC and HC groups. The results exhibited a significant separation between the ESCC and HC groups, indicating notable dysregulation in the plasma lipid profile of ESCC patients (Fig. 1A, Fig. 1B). The reliability of the PLS-DA result was further validated using a permutation test (n = 20) (Fig. 1C). Furthermore, based on the criteria of differential lipid (FDR < 0.05; VIP > 1.0; FC > 1.50 or FC < 0.667), a total of 5899 differential metabolic features were shown in the volcano plot, including 2654 up-regulated features and 3245 down-regulated features (Fig. 1D). |
| --- | --- | --- | --- | --- |
| Discussion | | | | |
| Key results | 18 | Summarise key results with reference to study objectives | 9 | The results revealed that 14 differentially expressed plasma lipids were associated with age (Supplemental Fig. S2), 14 ones with sex (Supplemental Fig. S3), 18 ones with smoking history (Supplemental Fig. S4), and only one with drinking history (Supplemental Fig. S5).  Compared the ESCC patients with and without lymph node metastasis, there were 11 differential lipids showed significant difference (P-value < 0.05), including FA 14:0, FA 15:4, FA 27:1, FA 28:7, FA 27:0, FA 42:0, DG 37:7, PC O-16:1_22:6, PC O-17:1_21:4, SM d18:1/23:0, PI 18:0_20:3. Among them, 6 were up regulation and 5 were down regulation in ESCC patients with lymph node metastasis. Boxplots illustrating the expression levels of these 11 lipids were shown in Figure 6. Out of the 99 differential lipids, 10 lipids—FA 14:0, FA 15:4, FA 28:7, FA 27:0, FA 28:0, DG 37:7, PE O-17:1_22:6, PE O-18:3_22:6, PC O-16:1_22:6, and SM d18:1/23:0—exhibited significant differences among the three stage groups (P-value < 0.05). Boxplots depicting the expression levels of these 10 lipids are shown in Figure 7. Among them, FA 15:4 displayed the most prominent upregulated trend in stage III compared to stages I and II, with the P-value of 7.27e-07. Only one of these 10 lipids, PE O-18:3_22:6, exhibited statistically significant differences in pairwise comparisons across stages I, II, and III, with an increase in expression levels in stage II but declining in stage III patients. |
| Limitations | 19 | Discuss limitations of the study, taking into account sources of potential bias or imprecision. Discuss both direction and magnitude of any potential bias | 10 | Limitation of the present study has to be mentioned. Firstly, the sample size was small and restricted to a single center. Additionally, a control group comprising high-risk individuals with benign lesions was not included. Moreover, lipid metabolism profiling analysis was not performed on serum or tissue samples, while only plasma samples were used to identify potential biomarkers. To improve future studies, it is recommended to increase the sample size, involve multiple centers, and incorporate a wider range of experimental samples from serum and tissue. |
| Interpretation | 20 | Give a cautious overall interpretation of results considering objectives, limitations, multiplicity of analyses, results from similar studies, and other relevant evidence | 10-11 | This study provides compelling evidence of significant dysregulation in the plasma lipid profile of ESCC patients compared to healthy controls (HC). A notable proportion of dysregulated lipids consisted of fatty acids (FA), diacylglycerols (DG), and triglycerides (TG). FA exhibited both upregulation and downregulation in ESCC patients. The observed upregulation of FA in the plasma can be partially attributed to the enhanced FA synthesis capacity of tumor cells, facilitated by increased expression and activity of key enzymes such as fatty acid synthase (FASN) (Currie et al. 2013). Consequently, certain FA concentrations in the plasma may be elevated. However, tumor cells may also increase their uptake and utilization of FAs, potentially leading to a decrease in FA concentration. The heightened metabolic activity of tumor cells promotes FA oxidation for energy production(Currie et al. 2013), further contributing to the lower FA concentration in the plasma. On the other hand, DG and TG showed a consistent downward trend in ESCC patients. DG and TG serve as primary forms of intracellular energy storage, and their downregulated concentrations in the plasma of ESCC patients may be linked to the heightened metabolic activity of tumor cells. The increased energy demands of rapidly proliferating tumor cells may result in the consumption of DG and TG through enhanced FA oxidation and energy utilization. This phenomenon may explain the observed downregulation of DG and TG levels in the plasma in this study. |
| Generalisability | 21 | Discuss the generalisability (external validity) of the study results | 11 | In conclusion, this study has developed a novel and valuable diagnostic model for ESCC by integrating plasma-based lipidomics and ML algorithms, enabling more efficient and accurate clinical diagnosis. Furthermore, the identified prognostic lipid markers exposed the dysregulated lipid metabolism in ESCC, which may provide new therapeutic targets to guide clinical treatment. In summary, this study has improved the understanding in the field of cancer diagnostic model construction by combining metabolomics and ML algorithms. This approach holds promise for cancer diagnosis and has the potential to promote the cancer treatment. |
| Other information | |  | | |
| Funding | 22 | Give the source of funding and the role of the funders for the present study and, if applicable, for the original study on which the present article is based | 11 | This research was supported by National Natural Science Foundation of China (No. 81672315,81302840); Medical and the Health Science Project of Zhejiang Province (2022KY622&2020KY487); Zhejiang Provincial Natural Science Foundation of China (LY23H010002); Key R&D Program Projects in Zhejiang Province (2018C04009). |

*Give information separately for cases and controls in case-control studies and, if applicable, for exposed and unexposed groups in cohort and cross-sectional studies.

**Note:** An Explanation and Elaboration article discusses each checklist item and gives methodological background and published examples of transparent reporting. The STROBE checklist is best used in conjunction with this article (freely available on the Web sites of PLoS Medicine at http://www.plosmedicine.org/, Annals of Internal Medicine at http://www.annals.org/, and Epidemiology at http://www.epidem.com/). Information on the STROBE Initiative is available at www.strobe-statement.org.
